# The international humanitarian response to famine in Tigray, Ethiopia:lessons from the Nigerian Civil War, 1967-1970

**DOI:** 10.1080/16549716.2022.2107203

**Published:** 2022-09-15

**Authors:** James F. Phillips, Charlotte M. Roy, Mulugeta Gebregziabher

**Affiliations:** a Heilbrunn Department of Population and Family Health, Mailman School of Public Health, Columbia University, New York, NY, USA; b Department of Emergency Medicine, Loma Linda University Medical Center, Loma Linda, CA, USA; c Department of Public Health Sciences, Medical University of South Carolina, Charleston, SC, USA

**Keywords:** Tigray, Ethiopia, Biafra, Nigerian Civil War, Nutritional emergency

## Abstract

The Tigray crisis in Ethiopia is a grave humanitarian catastrophe with causes and consequences that resemble the Nigerian Civil War that ended with the defeat of secessionist Biafra five decades ago. As in the Biafra example, an ethnically distinct and embattled enclave is surrounded by hostile forces and cut off from commerce of any kind, producing starvation, forced migrant encampments, and pervasive dependence on externally provided food relief. Relief action strategies developed during the Nigerian Civil War were comprised of operational components that were often insufficiently integrated into a unified system for nutritional screening, referral, acute care, nutritional rehabilitation, and team deployment. This lack of strategic integration for post-conflict relief actions merits review for possible lessons that could avert its recurrence in Tigray. If evidence-based systems for relief organization had been comprehensively applied in Biafra, the pace of post-conflict nutritional recovery could have been accelerated. Although component strategies of the Biafra-Nigeria Relief Action are being replicated by various agencies that are providing humanitarian assistance in Tigray, their collective impact could be enhanced if these strategies were integrated into a unified, evidence-driven systems response to the emergency. The elements of such a systems approach for assisting Tigray are reviewed.

## Background

Armed conflict between the Ethiopian military and the Tigrayan Defense Force (TDF) in northern Ethiopia has displaced over 1.8 million people [1]. A November 2020 decision of the Tigray People’s Liberation Front (TPLF) to proceed with regional elections, in defiance of a federal government-mandated postponement, was associated with a precipitous deterioration of relations between Tigrayan authorities and the federal government, culminating in an invasion of Tigray by the Ethiopian military in conjunction with incursions by Eritrean Defense Forces from the north and an Amhara regional militia from the south [2]. The combatants engaged in mass killings, sexual violence, looting, and destruction of farmlands [3]. Health facilities were destroyed [4], leading to a complete paralysis of the health system [5–7]. Accounts by international observers have asserted that areas of Western Tigray have been targeted for ‘ethnic cleansing [8,9].’ While Tigray has been more affected than other regions, human rights abuses have been instigated by all parties to the conflict, with adverse consequences that have affected neighboring regions of Ethiopia [10].

Ethiopia has emerged from this series of events as Africa’s epicenter of humanitarian crisis [11]. By mid-2021, estimates of Tigrayans experiencing catastrophic food insecurity exceeded 350,000, while an additional 5.2 million were estimated to be facing high levels of food insecurity [12], a crisis that is estimated to be worsening by 2022 [13,14].

While the origins of the Tigray conflict are unique, its general characteristics resemble the Nigerian Civil War that ended in January 1970 with the defeat of secessionist Biafra [15]. The histories, languages, and cultural traditions of territories comprising Biafra and Tigray contrast their respective heritages from that of other populations in their countries. Biafra’s unsuccessful secessionist war precipitated a famine that caused the death of at least 1.5 million people [16]. Like Biafra, Tigray is faced with famine as an embattled enclave cut off from critically needed supplies of any kind. These parallels invite questions about the strategic implications of humanitarian action during the Nigerian Civil War for the current response to famine in Tigray.

## Methods

The views expressed are based on personal field observations and experiences of the authors. James Phillips served during the Nigeria Civil War with the International Committee of the Red Cross (ICRC), May to July 1969, and subsequently with UNICEF as a ‘Forward Observer’ assigned to the Nigerian Red Cross (October 1969-June 1970) [17]. Charlotte Roy contributed personal observations to this essay based on her experience as a physician assigned to field operations of an emergency relief organization in Tigray in 2021. Mulugeta Gebregziabher contributed insights based on his ongoing survey research collaboration with Mekelle University faculty in Tigray that assessed the nutritional situation in the Tigray region [5]. While the authors have endeavored to maintain objectivity, they acknowledge that bias can be introduced by travel restrictions, communication limitations, and procedural restrictions that recur whenever armed conflict is the subject of observation [18].

## The Nigeria-Biafra relief action

Aid for Biafra was administered by an international consortium of 32 agencies [19]. The suffering of the Biafran population was globally publicized, with assistance limited to an airlift of food and medicine and emergency relief action support by humanitarian assistance teams [20]. Despite the nightly arrival of 30 or more cargo aircraft, the provision of relief supplies to the Biafran enclave was but a fraction of the supplies that were known to be needed [15,20,21]. Because the airlift was viewed by Nigerian authorities as an ‘act of war,’ its operation was limited to clandestine night flights [21,22]. International diplomacy throughout the war unsuccessfully sought to secure agreements for a land corridor for relief logistics operations. Despite the challenges incurred, the relief action undoubtedly saved many lives [20].

Less publicized at the time was aid provided to Nigerian-controlled areas of eastern Nigeria by agencies operating with terms of reference to the ICRC until July 1969 and the League of Red Cross Societies and the Nigerian Red Cross thereafter [23]. In the final months of 1969, massive stockpiles of commodity food supplies were accumulated and stored in Nigerian port warehouses. Relief teams conducted mass land-logistics supported food distribution to war affected areas, often in conjunction with nutritional screening, sickbay care, and rehabilitation [24]. Although this initiative failed to reach the population of the Biafran enclave during the conflict, it set the stage for supplying post-war rehabilitation efforts, as participating teams could transfer operations to the former Biafran enclave once hostilities ceased.

During the conflict, nutritional adversity in Biafra was so pervasive that priority was consigned to acute malnutrition therapy and direct food distribution to forced migrants [25]. Relief teams also attempted to provide mass distribution of food to the general population, although supplies were insufficient [22]. The situation in Nigerian military-controlled localities outside of the Biafran enclave was more conducive to organizing relief operations, because logistics challenges were less severe. The south-east sector of the Nigerian relief action was populated by ethnic groups who remained in their ancestral homeland when Nigerian military forces gained control of the locality, whereas ethnically Igbo people retreated into the Biafran heartland. In essence, the relief action became two separate operations by 1968, one for the Biafran enclave, the other for people residing in localities controlled by Nigerian forces.

During the conflict, lack of coordination among agencies was problematic. In Biafra, Caritas, protestant church organizations, and the Biafran Red Cross often operated independently. On the Nigerian side, independent operational management also impeded strategic integration, with some teams focusing on sickbay care, others on hospital care or food distribution to forced migration settlements, while some teams engaged in mass food distribution. This limitation of the Nigeria-Biafra relief operation was mitigated somewhat as the end of the conflict approached, because relief agencies in Nigerian-controlled areas could supplement their land-logistics capabilities with an integrated system of community-engaged nutritional screening, referral, and nutritional care and rehabilitation [17]. This strategic integration was inspired by innovations developed by the Quaker Service Team. The ‘Quaker Arm Circumference’ (QUAC) method was used to identify children at risk of malnutrition on the relationship between mid-upper arm circumference (MUAC) and height [26]. MUAC has since become a widely used nutritional screening tool [27]. In Quaker team served localities, QUAC device-based nutritional status survey research was used to identify areas in greatest need [28]. Within these priority areas, QUAC screening was used to identify children deemed to be in need of the controlled provision of supplemental cooked food. Volunteer outreach was used for conducting QUAC screening and referral operations, creating an objective community-based canvassing and referral system [26]

The Quaker system was adopted by several relief teams soon after the end of the conflict. However, population surveys were not uniformly applied for guiding team deployment or prioritizing commodity delivery locations, even when conditions permitted this approach. And, while emergency clinical support was effective at points of care, the objective application of screening criteria for community-engagement, referral, and recovery care was sporadic. Despite improved access to supplies when the conflict ended, a lack of adequate land logistics planning and action needlessly constrained supply flow volume.

There is a significant risk that such procedural inconsistencies could recur in Ethiopia when conflict subsides. Tigray will need a population-wide strategy that extends beyond its current focus on the supply of emergency relief to displaced people, in analogy to the systems approach eventually applied in post-war Nigeria.

## A systems approach for Tigray

Components of the systems approach pioneered in post-conflict Nigeria are currently employed in Tigray. Mass food distribution sponsored by the World Food Program (WFP) provided emergency food assistance to 620,000 people in Tigray between March and May 2022 [29]. Several humanitarian aid agencies are engaged in MUAC-based malnutrition screening and treatment of moderate and severe acute malnutrition. For example, the United Nations Office for the Coordination of Humanitarian Assistance (UNOCHA) reported that over 70,000 children under five were screened for malnutrition in June 2022, although this represents a relatively small number of children given the population at risk [14,30]. Greater than one in five of the children screened were found to be malnourished.

Despite this inter-agency humanitarian commitment to Tigray, humanitarian efforts have been hindered significantly by supply delivery barriers. Although the withdrawal of Ethiopian military in June 2021 improved prospects for accessing rural areas within Tigray, the provision of supplies to the region was virtually halted by the destruction of multiple bridges at the time of the government’s departure. Movement of goods into landlocked Tigray from Eritrean ports has also been infeasible owing to that country’s political alignment with the Federal Government of Ethiopia [31]. The WHO Director-General characterized the situation as a ‘blockade’ on humanitarian assistance to Tigray [32]. In recent months shipments of humanitarian supplies have nonetheless resumed following a truce announced by the Ethiopian Government [29]. Yet, distribution of supplies within Tigray remains challenging due to fuel shortages and insecurity [30] Relief agency strategic coordination is a continuing challenge that has been exacerbated by Government of Ethiopia decision to suspend the activities of several organizations [33].

Although the provision of emergency assistance to displaced persons must be sustained, post-conflict planning is urgently needed. Tigray is at risk of catastrophic famine if logistics problems, rehabilitation needs, and team coordination challenges are not addressed. The dire situation in Tigray invites reflection on ways that the life-saving potential of relief action components could be implemented as a unified system of care within a common strategic framework.

There is a possibility that post-conflict expansion of team deployment for Tigray could lead to a recurrence of incomplete systems integration that diminished the efficacy of humanitarian action in Nigeria: Interlinked land and air logistics, population-based surveillance, screening, and referral for acute care and supplemental feeding were elements of a generalized system of care for Biafra that failed to take shape until the crisis was nearly over. To be fully effective, humanitarian support for Tigray will require systems integration. Priority currently consigned to the provision of full nutritional support for displaced populations must continue. However, if the logistical barriers to the distribution of food can be resolved throughout the region, an evidence-grounded strategy could become possible that would extend operations to vulnerable individuals in the general population. Existing community-based primary health-care capabilities could underpin such a system of care. Prior to the conflict, Tigray had a strong community-based primary health-care system known as the Health Extension Program (HEP) [34,35]. However, HEP disintegrated with the onset of the conflict. International support for post-conflict health development should be directed to reconstituting HEP. Irrespective of whether Tigray achieves sovereignty or remains part of the state of Ethiopia, HEP could provide a platform for the rapid development of a six component systems approach to nutritional care and rehabilitation [4]:

- Populations comprised of forced migrants require mass distribution of uncooked commodity food. However, a MUAC population-based sample survey appraisal of all Tigray localities could define priority areas for team assignment and mass commodity distribution and clarify relative levels of severity of need in localities known to require support.
- Community-based appraisal operations could be designed to provide continuous information on the epidemiology of nutritional adversity as well as evidence-driven screening and referral.
- This application of evidence-based screening and referral within priority localities could facilitate the targeting of resources on children in greatest need.
- The involvement of community mobilization in the screening and referral process could engage local leaders in sustaining screening and referral operations, obviating the need for widespread international team deployment, while enabling external support to be targeted on acute care.
- Current operations in Tigray prioritize acute care needs, but if links to a screening and referral operation could be added, this would enhance the life-saving potential of clinical interventions. In particular, the currently disrupted system of hospitals and clinics for tertiary care must be reconstituted as the apex of the referral system.
- Children who have recovered from acute adversity could be monitored and supported with the provision of ready-to-use therapeutic food, employing MUAC as a community-implemented monitoring and referral tool.

## Conclusion

The humanitarian crisis in Ethiopia will be challenging for aid agencies to address until its underlying political causes are resolved [36]. Yet, the resolution of post-conflict organizational challenges in Ethiopia in the 1980s [37], and elsewhere [38], demonstrate that strategic planning for post-conflict Tigray and surrounding regions is urgently needed. The lack of comprehensive systems integration in Nigeria hindered the effectiveness of relief operations, not only during the conflict but also upon the war’s termination. There is a risk that Biafra’s history of dysfunctional planning could recur in Ethiopia, with millions of lives at stake. Applying the systems integration lessons from post-conflict Nigeria to Tigray could help avert such a catastrophe.

## Context

Forced migration and food insecurity are mounting public health problems in Africa. The current Ethiopian Tigray conflict represents the most pressing such catastrophe. Its causes and consequences resemble the Nigerian Civil War that ended with the defeat of secessionist Biafra in 1970. Biafra-Nigeria relief action strategies were inadequately integrated into a systems approach to nutritional rehabilitation, diminishing its life-saving potential. Strategic planning for Tigray is needed to enable relief operations to avoid this pitfall.
